# Systematic Review and Meta-Analysis of Efficiency and Safety of Double-Lumen Tube and Bronchial Blocker for One-Lung Ventilation

**DOI:** 10.3390/jcm12051877

**Published:** 2023-02-27

**Authors:** Piotr Palaczynski, Hanna Misiolek, Lukasz Szarpak, Jacek Smereka, Michal Pruc, Mateusz Rydel, Damian Czyzewski, Szymon Bialka

**Affiliations:** 1 Department of Anaesthesiology and Intensive Care, Faculty of Medical Sciences in Zabrze, Medical University of Silesia, 41-800 Zabrze, Poland; 2 Henry JN Taub Department of Emergency Medicine, Baylor College of Medicine, Houston, TX 77030, USA; 3 Research Unit, Maria Sklodowska-Curie Bialystok Oncology Center, 15-027 Bialystok, Poland; 4 Department of Emergency Medical Service, Wroclaw Medical University, 51-616 Wroclaw, Poland; 5 Research Unit, Polish Society of Disaster Medicine, 05-806 Warsaw, Poland; 6 Department of Thoracic Surgery, Faculty of Medical Sciences in Zabrze, Medical University of Silesia, 41-800 Zabrze, Poland

**Keywords:** double-lumen tube, DLT, bronchial blocker, one-lung ventilation, safety, airway management, meta-analysis

## Abstract

One-lung ventilation is also used in some thoracic or cardiac surgery, vascular surgery and oesophageal procedures. We conducted a search of the literature for relevant studies in PubMed, Web of Science, Embase, Scopus and Cochrane Library. The final literature search was performed on 10 December 2022. Primary outcomes included the quality of lung collapse. Secondary outcome measures included: the success of the first intubation attempt, malposition rate, time for device placement, lung collapse and adverse events occurrence. Twenty-five studies with 1636 patients were included. Excellent lung collapse among DLT and BB groups was 72.4% vs. 73.4%, respectively (OR = 1.20; 95%CI: 0.84 to 1.72; *p* = 0.31). The malposition rate was 25.3% vs. 31.9%, respectively (OR = 0.66; 95%CI: 0.49 to 0.88; *p* = 0.004). The use of DLT compared to BB was associated with a higher risk of hypoxemia (13.5% vs. 6.0%, respectively; OR = 2.27; 95%CI: 1.14 to 4.49; *p* = 0.02), hoarseness (25.2% vs. 13.0%; OR = 2.30; 95%CI: 1.39 to 3.82; *p* = 0.001), sore throat (40.3% vs. 23.3%; OR = 2.30; 95%CI: 1.68 to 3.14; *p* < 0.001), and bronchus/carina injuries (23.2% vs. 8.4%; OR = 3.45; 95%CI: 1.43 to 8.31; *p* = 0.006). The studies conducted so far on comparing DLT and BB are ambiguous. In the DLT compared to the BB group, the malposition rate was statistically significantly lower, and time to tube placement and lung collapse was shorter. However, the use of DLT compared to BB can be associated with a higher risk of hypoxemia, hoarseness, sore throat and bronchus/carina injuries. Multicenter randomized trials on larger groups of patients are needed to draw definitive conclusions regarding the superiority of any of these devices.

## 1. Introduction

Several procedures used in various types of surgery require general anaesthesia with one-lung ventilation; these include procedures used mainly in thoracic surgery, including increasingly using minimally invasive techniques, among them video-assisted thoracoscopic surgery (VATS) and cardiac surgery, particularly including minimally invasive cardiac surgery (MICS), which is carried out using the mini-thoracotomy method [1,2]. One-lung ventilation is also used in some thoracic, vascular surgery and oesophageal procedures [3,4]. Increasingly, minimally invasive techniques are being used, which have many benefits for patients, but where reliable one-lung ventilation (OLV) is essential. During the COVID-19 pandemic, attempts were also made to ventilate both lungs independently using double-lumen intubation techniques [5,6].

Of particular importance are thoracic surgeries, which are related to the specificity of anaesthetic management, including the need to provide ventilation of one lung with a properly collapsed lung, which is achieved by using special methods of airway management, including the use of special endotracheal tubes and/or bronchial blockers [7,8,9]. A specific feature of thoracic surgery is the need for one-lung ventilation to, among other factors, ensure good conditions in the surgical field and facilitate surgical exposure [10].

Double-lumen tubes can be of the Robertshaw double-lumen tube (DLT) type. DLTs are considered the gold standard for airway management for procedures in patients with one-lung ventilation [11,12]. This type of tube has the advantages of reliably and quickly obtaining one-lung ventilation and excellent airway suction capabilities. This tube allows bronchoscopy and is characterized by a low price.

An alternative to DLT is the use of bronchial blockers. There are both Univent tubes, which are single-lumen tubes with bronchial blocker system, which is also used in EZ Blocker tubes, and independent free-standing bronchial blockers, which are used with classic single-lumen tubes (Arndt Endobronchial Blocker System) as well as The Cohen Flex-tip Blocker, Uniblocker or Coopdech blocker. Magill described the first use of a bronchial blocker in 1936 [13].

The results of studies to date do not indicate the superiority of one technique over the other, while the number of studies and study groups is limited. Given the serious clinical choice in one-lung ventilation, it is essential to analyze the overall results.

The purpose of this study is to perform a meta-analysis of studies comparing one-lung ventilation using double-lumen tubes and bronchial blockers.

## 2. Materials and Methods

### 2.1. Protocol and Registration

This meta-analysis was conducted and reported following the Preferred Reporting Items for Systematic Reviews and Meta-Analyses (PRISMA) statement [14] and was registered with PROSPERO prior to completion of the initial search (registration No: CRD42022382135).

### 2.2. Eligibility Criteria

Studies that were included in this meta-analysis had to fulfil the following PICOS criteria: (1) Participants, patients 18 years old or older under general anaesthesia and single lung ventilation; (2) Intervention, airway management with double lumen tube; (3) Comparison, airway management with brachial blocker; (4) Outcomes, time for device placement, time for lung collapse, quality of lung collapse, malposition rate, the success rate of first intubation attempt and adverse events occurrence; (5) Study design, randomized controlled trials comparing DLT and BB for airway management and one-lung ventilation.

Exclusion criteria were studies only reporting on one airway management technique (DLT or BB) or studies reporting DLTs with a camera on the tip. We also excluded studies conducted on animals or pediatric patients (under 18 years old) and articles in languages other than English and article design such as reviews, editorials, letters, conferences and meetings abstracts or articles that do not contain original data.

### 2.3. Data Sources and Searches

PubMed, Web of Science, Embase, Scopus and Cochrane Library were searched independently by two reviewers (P.P. and M.P.) for clinical trials comparing DLT and BB. When the preliminary conclusions were uncertain, the literature was reassessed by all of the authors. All databases were searched from inception, and the last search date was 10 December 2022. A specific and appropriate search strategy was used for each database. We used the following search terms: “Double lumen tube” OR “dual lumen tube” OR “DLT” AND “bronchial blocker” AND “thoracic surgery” OR “one-lung ventilation” OR “lung isolation”. All references were imported into Endnote version X9 (Thomson Reuters, Toronto, ON, Canada), and duplicates were removed before exporting them to the software-screening tool, Rayyan [15].

### 2.4. Data Extraction and Quality Assessment

Two reviewers (P.P. and M.P.) extracted data independently using the predefined form. Potential disagreement arose data extraction was resolved through a discussion with another reviewer (S.B.). From each study, data were extracted on: (A) study characteristics (i.e., name of the first author, year of publication, inclusion and exclusion criteria, the primary outcome(s), findings); (B) patient characteristics (i.e., population, male gender, age, body mass index, ASA score, Mallampati classifications, type of surgery); (C) intubation outcomes (i.e., first intubation attempt success rate, quality of lung collapse (excellent, fair, poor), malposition rate, times for lung device placement and lung collapse, adverse events (hypoxemia, hoarseness, sore throat and lung infection).

Two reviewers (M.P. and A.D.) independently assessed the individual studies for risk of bias. In the event of discrepancies in the assessment by the above reviewers, all authors performed the quality assessment again. For each study, the risk of bias was assessed at the study level using the Rob2 tool (A revised tool to assess the risk of bias in randomized trials) for randomized trials [16] and ROBINS-I (Risk Of Bias In Non-randomized Studies—of Interventions) bias assessment tool for non-randomized studies [17]. The Robvis application was used to visualize the risk of bias assessments [18].

### 2.5. Outcome Measures

The primary outcome measure was the quality of lung collapse, defined as excellent, fair, or poor. Secondary outcome measures included: malposition rate, time for device placement and lung collapse and adverse events occurrence.

### 2.6. Statistical Analysis

All analyses were conducted using the RevMan 5.4 software (Cochrane Collaboration, London, UK). For binary outcomes, odds ratios (OR) with 95% confidence intervals (CI) were calculated. For continuous outcomes, we used mean differences (MDs) as the effect measure with 95%CI. If outcomes were reported as median with interquartile range, using a Hozo formula [19], means and standard deviations were estimated. Cochran’s Q test and Higgins I2 statistic method were used to test heterogeneity, with 25%, 50% and 75% considered moderate, substantial and considerable heterogeneity, respectively [20]. The random-effect model was used when heterogeneity was significant (I^2^ > 50%). Otherwise, the fixed-effect model was applied. A 2-tailed *p* < 0.05 was considered statistically significant for all analyses. Testing for publication bias was evaluated visually by the funnel plot and further assessed using the Egger test of asymmetry applied to the funnel plot. Due to substantial heterogeneity, we did not adjust for publication bias [21]. Additionally, we did a sensitivity analysis to investigate each study’s influence on the overall results by omitting each from the meta-analysis [22].

## 3. Results

### 3.1. Study Selection

**Figure 1:**
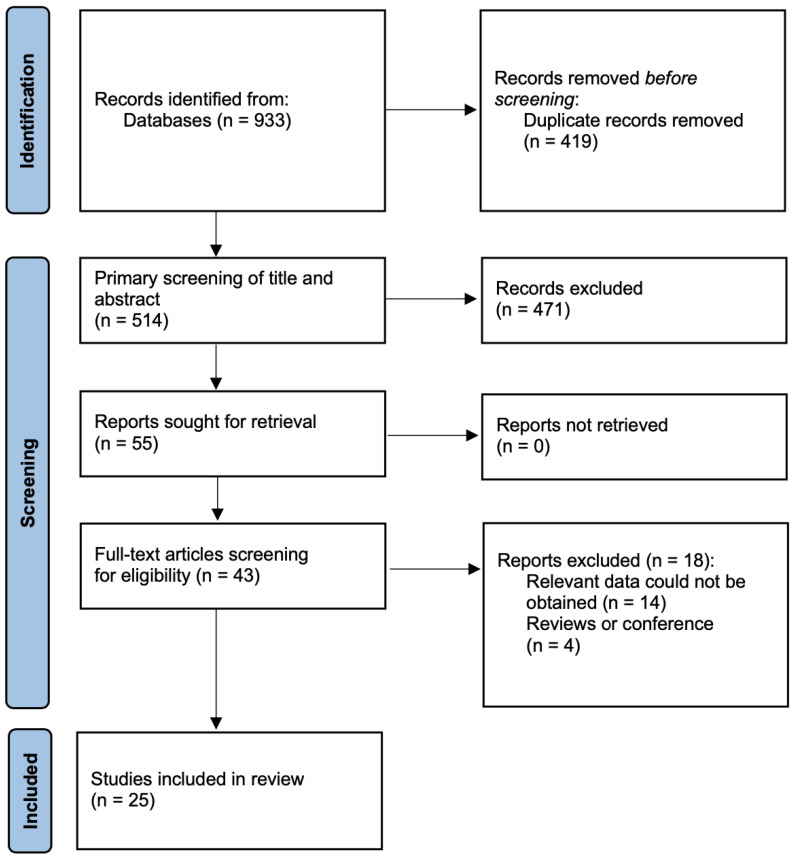
Flow chart of the literature search and selection.

The outline of the study selection process is depicted in a PRISMA diagram (Figure 1). Our search yielded 933 results, of which 419 were duplicates and were removed. We screened the remaining 514 titles and abstracts, excluding 471 studies that did not fulfil our inclusion criteria. The full text was read from 43 articles. Finally, 25 studies published between 1996 and 2022 were included in this meta-analysis [10,13,23,24,25,26,27,28,29,30,31,32,33,34,35,36,37,38,39,40,41,42,43,44,45]. A total of 1636 patients were evaluated across the 25 studies, with 740 patients in the double-lumen tube group and 896 in the bronchial blocker group.

### 3.2. Study Characteristics

The baseline characteristics of included trials are presented in Table 1. The study sample size ranged from 28 to 160 patients. Of the 25 trials, 8 were conducted in China [10,24,26,37,38,41,44,45], 6 in the USA [25,29,30,31,32,34], 2 in Canada [13,36], 2 in Germany [33,46], 2 in Korea [42,43], 2 in France [23,27], and 1 study each in the following countries: Austria [39], Egypt [40] and the Netherlands [35]. Pooled analysis of patients’ characteristics was presented in Supplementary Table S1. The 25 included studies were all randomized controlled studies, so there was a low risk of hidden bias (Supplementary Figures S1 and S2).

### 3.3. Meta-Analysis

**Figure 2:**
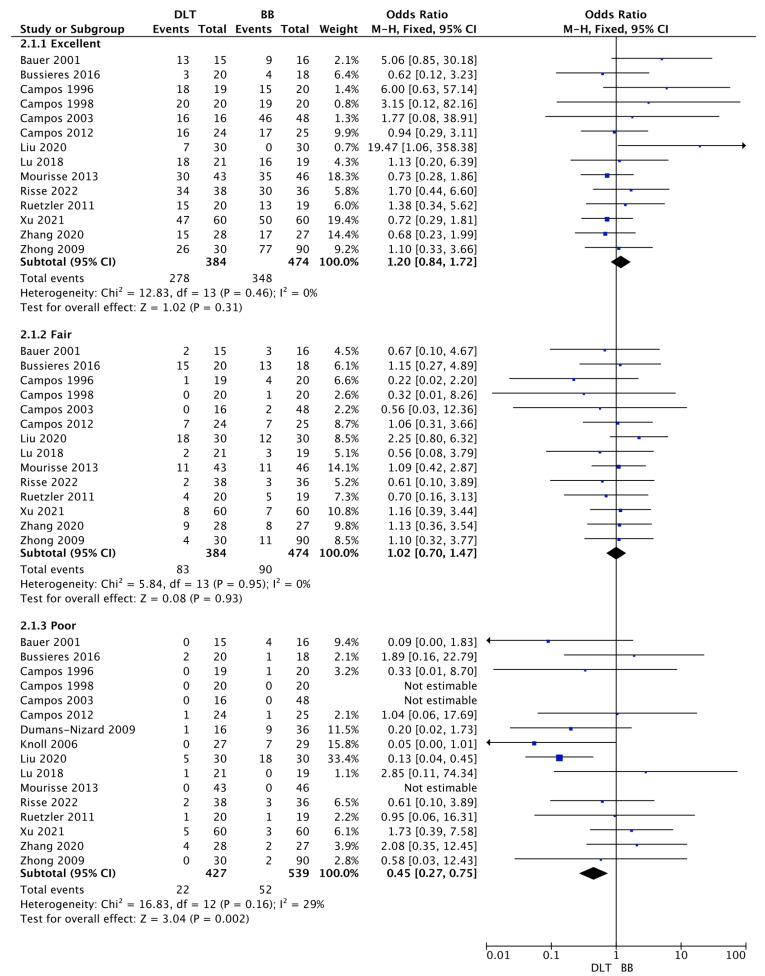
Forest plot of quality of lung collapse among double lumen tube (DLT) and bronchial blocker (BB) groups. The centre of each square represents the odds ratios for individual trials, and the corresponding horizontal line stands for a 95% confidence interval. The diamonds represent pooled results [23,24,25,26,27,28,29,30,31,33,35,39,41,44,45,46]. Legend: CI: confidence interval.

Pooled analysis of the quality of lung collapse is presented in Figure 2.

Fourteen studies reported excellent lung collapse among DLT and BB groups, 72.4% vs. 73.4%, respectively (OR = 1.20; 95%CI: 0.84 to 1.72; *p* = 0.31). There were also no statistically significant differences in the quality of fair lung collapse (21.6% vs. 19.0%; OR = 1.02; 95%CI: 0.70 to 1.47; *p* = 0.93). However, the use of DLT was associated with a statistically significantly lower risk of poor lung collapse compared to BB (5.2% vs. 9.6%, respectively; OR = 0.45; 95%CI: 02.7 to 0.75; *p* = 0.002).

**Figure 3:**
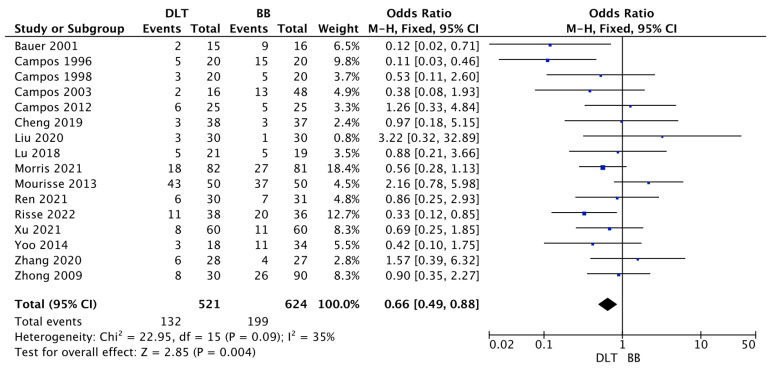
Forest plot of malposition rate among double lumen tube (DLT) and bronchial blocker (BB) groups. The centre of each square represents the odds ratios for individual trials, and the corresponding horizontal line stands for a 95% confidence interval. The diamonds represent pooled results [10,24,25,26,27,29,30,31,34,35,38,41,42,44,45,46]. Legend: CI: confidence interval.

The malposition rate was reported among sixteen trials. Pooled analysis of malposition in DLT and BB varied and amounted to 25.3% vs. 31.9%, respectively (OR = 0.66; 95%CI: 0.49 to 0.88; *p* = 0.004; Figure 3).

Pooled analysis of adverse events is presented in Table 2. The use of DLT compared to BB was associated with a higher risk of hypoxemia (13.5% vs. 6.0%, respectively; OR = 2.27; 95%CI: 1.14 to 4.49; *p* = 0.02), hoarseness (25.2% vs. 13.0%; OR = 2.30; 95%CI: 1.39 to 3.82; *p* = 0.001), sore throat (40.3% vs. 23.3%; OR = 2.30; 95%CI: 1.68 to 3.14; *p* < 0.001), and bronchus/carina injuries (23.2% vs. 8.4%; OR = 3.45; 95%CI: 1.43 to 8.31; *p* = 0.006).

**Figure 4:**
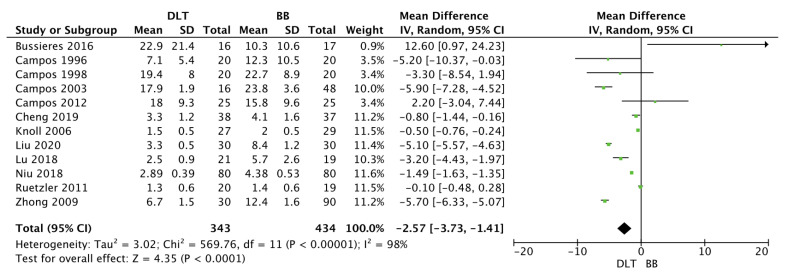
Forest plot of time to lung collapse (min) among double lumen tube (DLT) and bronchial blocker (BB) groups. The centre of each square represents the mean differences for individual trials, and the corresponding horizontal line stands for a 95% confidence interval. The diamonds represent pooled results [10,24,25,26,28,29,30,31,33,37,39,45].

**Figure 5:**
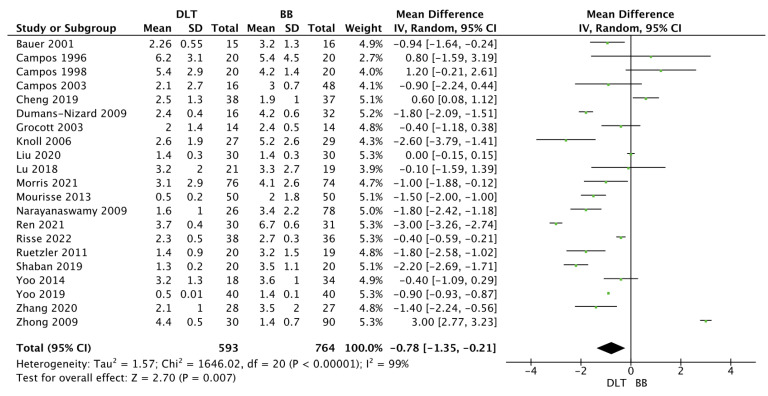
Forest plot of time to tube placement (min) among double lumen tube (DLT) and bronchial blocker (BB) groups. The centre of each square represents the mean differences for individual trials, and the corresponding horizontal line stands for a 95% confidence interval. The diamonds represent pooled results [10,23,24,25,26,27,29,30,32,33,34,35,36,38,39,40,42,43,44,45,46].

Time to device placement in the DLT group was 2.5 ± 2.1 min, compared to 3.1 ± 2.1 min in the BB group (MD = −0.78; 95%CI: −1.35 to −0.21; *p* = 0.007; Figure 4). Time for lung collapse in DLT and BB groups varied and amounted to 7.0 ± 8.9 vs. 10.3 ± 8.3 min, respectively (MD = −2.57; 95%CI: −3.73 to −1.41; *p* < 0.001; Figure 5).

Duration of surgery was indicated in seven trials and was 156.3 ± 77.1 min for DLT, compared to 146.5 ± 70.7 min for BB (MD = 3.17; 95%CI: −4.83 to 11.18; *p* = 0.44). In turn, the duration of anaesthesia was 198.8 ± 98.3 vs. 190.3 ± 82.9 min, respectively (MD = 5.00; 95%CI: −0.25 to 10.26; *p* = 0.06). Only five trials reported the duration of one-lung ventilation, which was 137.9 ± 76.9 min for DLT and 144.9 ± 99.9 min for the BB group (MD = −5.72; 95%CI: −41.40 to 29.96; *p* = 0.75).

## 4. Discussion

The studies conducted so far on comparing DLT and BB are ambiguous. The authors of most papers point to the ease of insertion of DLT tubes and faster insertion time. In favour of BB, there are fewer complications and the severity of these complications—mainly airway injuries. Either method works best in specific clinical scenarios (paediatrics, difficult airways, “dirty procedures”—large amounts of secretions obturating the BB lumen). As the results are inconclusive, while the benefits are particularly apparent in selected groups of patients, anesthesiologists should be familiar with and use both techniques.

Classic double-lumen tubes are equipped with a carinal hook that allows the tube to be properly positioned at the height of the carina [47]. However, modified versions without this element are also used, while these tubes have yet to be shown to have a higher malposition rate. One problem with the use of DLTs is the potential risk of hook amputation during insertion of the tube into the airway, which, however, rarely occurs in clinical practice, as well as several potential injuries caused by rotation of a tube equipped with a carinal hook, including mucosa injury [23].

Unfortunately, double-lumen tubes are more rigid compared to classic single-lumen tubes, leading to greater difficulty during intubation and a greater risk of complications associated with their insertion and holding in place [46]. Disadvantages of DLT include the need for bronchoscopy for positioning, complications associated with placement and the higher incidence of having to reattempt laryngoscopy during intubation with this tube [23]. Due to their specific design, using these tubes can cause injuries to the larynx and malposition, problems with difficult airways and abnormal tracheobronchial anatomy [23]. These problems arise from the size of DLTs and the need for rotation during their insertion within the airway, increasing the risk of injury. A potential problem with DLTs is the excessive cardiovascular response during intubation, compared to intubation with classic single-lumen tubes, which is essential in patients with cardiovascular disease [24]. This is particularly important in patients undergoing thoracic and cardiac surgery, as cardiac risk is elevated in these patients.

Although double-lumen tubes are available as right-sided and left-sided DLTs for anatomical reasons, mainly the right upper bronchus outlet, right-sided DLTs are rarely used; in the vast majority of cases, anesthesiologists use left-sided DLTs [48]. The problem with right-sided DLTs is the potential for obstruction of the right upper bronchus and poor-quality lung collapse of the right upper lobe. These problems can also apply to right-sided bronchial blockers [25,49].

The problem with bronchial blockers is the degree of pressure on the airway mucosa during prolonged surgery and the pressure in the bronchial blocker cuff [24]. The advantages of bronchial blockers are that they can be used with a conventional endotracheal tube without needing re-intubation after the procedure. They can be used in difficult airways and pediatric patients with fewer laryngoscopy attempts [50,51]. Disadvantages include a higher price, the need for bronchoscopy to position most models, and, more often than not, poorer quality of lung collapse in terms of surgical field conditions [52]. The choice of BB should also consider specific situations mainly related to thoracic surgery, including empyema, hemothorax, and the presence of secretions and blood in the trachea and bronchus, which is associated with risks to the healthy lung [26].

The higher incidence of complications associated with the DLT tube is most likely due to the physical properties (larger, more rigid) and the insertion technique (90-degree rotation) [24]. The risk of displacement is significant due to the possibility of hypoxia, even interruption of the procedure and the possibility of airway injury [53].

The cost of purchasing a bronchial blocker is significantly higher than double-lumen tubes. In the analysis of the total cost of the procedure, this difference does not fundamentally affect the choice of equipment, but it is noticeable when accurately counting anaesthesia costs. In the analysis of the equipment costs, however, differences in the risk of complications and the length of the procedure should be taken into account, which in some cases may compensate for the higher cost of the bronchial blocker. New DLT tube solutions are emerging, where perhaps the need for FB-VDLT and ANKOR-DLT will be eliminated, which may give some advantages in terms of economics.

In this analysis, we considered the primary elements associated with the use of DLT and BB, including the quality of lung collapse score, rated excellent, fair and poor and also time for lung collapse and time for device placement (min) as well as malposition rate Adverse events including hypoxemia, hoarseness, sore throat and lung infections.

Quality of lung collapse is essential for performing thoracic surgery, including notably VATS procedures conditions of the surgical field are fundamental to the surgeon’s ability to perform the procedure and the risk of complications. For optimal conditions, sufficient lung collapse must occur, which can be rated on a simple scale as excellent or fair, as opposed to challenging conditions with poor quality of lung collapse rated as poor. One of the factors affecting the quality of lung collapse may be the use of the VTS technique with CO_2_ insufflation.

Regarding the quality of lung collapse rated excellent and fair, there were no statistically significant differences between the analyzed devices, with this parameter evaluated in 14 studies. However, the evaluation of poor was statistically significantly more frequent for BB. However, it is essential to note the differences in how the quality of lung collapse was assessed between studies and the lack of assessment standards. Lung collapse after the isolation of one lung occurs in two phases. In the first phase, there is a relatively rapid lung collapse.

In contrast, the second phase is slower, associated with small airway closure and residual lung gases, and depends on atelectasis due to lung gas exchange [10]. Some studies have raised questions about the reliability of the surgeon’s assessment of the quality of lung collapse, suggesting that a methodologically better approach would be to analyze the video recording of the procedure rather than relying on the subjective assessment of the operator. The operator’s awareness of the type of airway equipment used (DLT vs. BB) when assessing the quality of lung collapse during the procedure can also raise a methodological problem.

The quality of lung collapse is also affected by the surgical technique used. With thoracotomy, unlike the VATS technique, the operator can increase the degree of lung deflation by direct manual lung compression or by using a lung retractor [34].

Time for lung collapse (min) was analyzed in 12 studies and was statistically significantly shorter for DLT, 9.8 vs. 12.3 min, while the difference reached 2.5 min. The authors cite various explanations for the differences in time for lung collapse using DLT and BB. DLTs have a larger diameter than BBs, potentially resulting in a lower risk of gas leakage and providing a faster time for lung collapse with DLTs. This may affect the duration of the entire procedure [34]. The heterogeneity of techniques to achieve lung collapse, including the type of disconnection technique when using BBs, may also influence the result.

Time-to-device placement was analyzed in 21 studies and was found to be shorter for DLT (2.5 vs. 3.2 min).

Malposition is a common problem during one-lung ventilation procedures. The problem results from the improper fixation of the tube itself or the blocker and changes in patient position and movement of anatomical elements during the procedure. The malposition rate was analyzed in 16 studies and was relatively high for both devices, but malposition was more common with BB at 31.9% vs. 25.3%. Attention should be paid to the effect of the patient’s body position during surgery and changes in this position on the malposition rate, especially changes in the patient’s head position and also changes in the patient’s position from supine position to lateral decubitus position and surgical manipulations within the lung [29]. The correct position of the tube or blocker significantly impacts the optimal degree of lung collapse and the reduction of perioperative complications. Some investigators suggest mandatory rechecking of DLT or BB position with fiberoptics after each change in a patient position [29].

Hypoxemia during surgery is a severe risk to the patient and can cause further serious complications [53]. Hypoxemia was analyzed in six studies and occurred in 13.5% of patients in the DLT group and 6.0% of patients in the BB group.

Injuries during the insertion or removal of DLTs and BBs can cause hoarseness and sore throat, among other factors, whose resolution time varies and can affect the quality of anaesthesia from the patient’s perspective.

Hoarseness was analyzed in nine studies and occurred in 25.2% of patients in the DLT group and only 13.0% in the BB group. Postoperative hoarseness is associated with various factors such as intubation technique, duration of surgery, type of surgery and endotracheal tube size, and patient-related factors such as gender [33].

The sore throat was analyzed in 12 studies; it occurred more frequently in the DLT group (40.3%) and (23.3%) in the BB group. The larger size, stiffness and diameter of the DLT can explain the higher incidence of sore throat, a significant clinical problem affecting quality assessment from the patient’s perspective, and sore throat can persist for days after the procedure. However, it should be considered that sore throat frequently occurs in patients after general anaesthesia with intubation with classic single-lumen tubes [35].

Lung infection was analyzed in only three studies, with no statistically significant differences between the DLT and BB groups. There are many concerns about the risk of healthy lung infection during one-lung ventilation. However, the results obtained regarding this type of airway management device are inconclusive.

It should be noted that in our analysis, factors such as duration of surgery, length duration of anaesthesia and duration of one lung ventilation did not affect in any way due to the lack of statistical significance, which emphasizes the lack of influence of these factors on complications in patients using individual devices.

### Limitations

The main limitations of the analysis include the inclusion of different types of airway management equipment, including a variety of studies involving different types of double-lumen tubes and bronchial blockers. Most studies used left-sided DLTs and BBs; however, some used left-sided and right-sided. Limitations also include variation in blinding regarding the methods used and the nature of the interventions. Variation in the experience of anesthesiologists regarding the methods used for airway management is also a major limitation. The heterogeneity of the included studies, including the inclusion of VATS procedures and thoracotomy procedures, is also a limitation in the analyzed papers. In most cases, the studies analyzed were single-centre. Limitations also include the heterogeneity of the groups of patients studied, including the exclusion of some patients with selected thoracic pathologies in some analyses. In contrast, others included selected pathologies, such as morbidly obese patients. Other limitations include the small number of studies, including small study groups, and gender bias.

## 5. Conclusions

The studies conducted so far on comparing DLT and BB are ambiguous. Regarding the quality of lung collapse, rated excellent and fair, there were no statistically significant differences between the analyzed devices. However, the evaluation of poor was statistically significantly more frequent for BB. In the DLT compared to the BB group, the malposition rate was statistically significantly lower, and time to tube placement and lung collapse was shorter. The use of DLT compared to BB can be associated with a higher risk of hypoxemia, hoarseness, sore throat and bronchus/carina injuries. Multicenter randomized trials on larger groups of patients are needed to draw definitive conclusions regarding the superiority of any of these devices.

## Figures and Tables

**Table 1:** Baseline characteristics of included trials.

Study	Country	Double-Lumen Tube Group					Bronchial Blocker Group				
		No. of Patients	Age	Sex, Male	BMI	ASA I–II	No. of Patients	Age	Sex, Male	BMI	ASA I–II
Bauer et al., 2001 [27]	France	16	NS	NS	NS	NS	19	NS	NS	NS	NS
Bussieres et al., 2016 [28]	Canada	20	63 ± 11	9 (45.0%)	27.9 ± 6.1	NS	18	62 ± 8	8 (44.0%)	28.3 ± 5.1	NS
Campos et al., 1996 [29]	USA	20	NS	NS	NS	NS	20	NS	NS	NS	NS
Campos et al., 1998 [25]	USA	20	NS	NS	NS	NS	20	NS	NS	NS	NS
Campos et al., 2003 [30]	USA	16	NS	NS	NS	NS	48	NS	NS	NS	NS
Campos et al., 2012 [31]	USA	25	NS	NS	39.9 ± 4.8	NS	25	NS	NS	40.2 ± 4.5	NS
Cheng et al., 2019 [10]	China	38	51.1 ± 7.3	26 (68.4%)	24.2 ± 3.1	38 (100%)	37	53.2 ± 9.1	24 (64.9%)	23.4 ± 4.3	37 (100%)
Dumans-Nizard et al., 2009 [23]	France	16	63 ± 3.5	11 (68.8%)	NS	NS	32	58.8 ± 4.8	22 (68.8%)	NS	NS
Grocott et al., 2003 [32]	USA	14	56 ± 14	NS	NS	NS	14	62 ± 12	NS	NS	NS
Knoll et al., 2006 [33]	Germany	27	60.4 ± 8.5	17 (63.0%)	NS	NS	29	62.8 ± 8.5	17 (58.6%)	NS	NS
Liu et al., 2020 [26]	China	30	55.5 ±11.3	17 (56.7%)	23.6 ± 4.2	27 (90.0%)	30	56.5 ± 14.5	14 (46.7%)	21.8 ± 8.9	26 (86.7%)
Lu et al., 2018 [24]	China	21	66 ± 6	16 (76.2%)	23 ± 3	14 (66.7%)	19	68 ± 9	13 (68.4%)	22 ± 2	14 (73.7%)
Morris et al., 2021 [34]	USA	37	66.2 ± 12.9	25 (67.6%)	28.3 ± 4.79	0 (0.0%)	38	62.1 ± 10.5	21 (55.3%)	27.8 ±4.8	0 (0.0%)
Mourisse et al., 2013 [35]	Netherlands	50	59 ± 13.6	35 (70.0%)	NS	NS	50	61 ± 13.3	36 (72.0%)	NS	NS
Narayanaswamy et al., 2009 [36]	Canada	26	NS	NS	26.7 (4.2)	NS	78	NS	NS	28 ± 6	NS
Niu et al., 2018 [37]	China	80	44.21 ± 5.14	48 (60.0%)	NS	NS	80	43.34 ± 4.28	44 (55.0%)	NS	NS
Ren et al., 2021 [38]	China	30	52.5 ± 3.4	10 (33.3%)	22.9 ± 2.5	NS	31	52.5 ± 5.3	13 (41.9%)	22.8 ± 2.2	NS
Risse et al., 2022 [46]	Germany	38	64.3 ± 4.8	25 (65.8%)	25 ± 1.9	9	36	64.8 ± 3.3	23 (63.9%)	26.9 ± 1.6	7 (19.4%)
Ruetzler et al., 2011 [39]	Austria	20	61.9 ± 14.4	12 (60.0%)	NS	NS	19	54.4 ± 20.2	8 (42.1%)	NS	NS
Shaban et al., 2019 [40]	Egypt	20	41.7 ± 9.3	7 (35.0%)	26.68 ± 6.75	20 (100%)	20	42.4 ± 8.5	12 (60.0%)	27.26 ± 5.64	20 (100%)
Xu et al., 2021 [41]	China	60	51.9 ± 11.9	34 (56.7%)	23.2 ± 1.9	55 (91.7%)	60	62 ± 6.2	32 (53.3%)	22.2 ± 5.7	55 (91.7%)
Yoo et al., 2014 [42]	Korea	18	20.8 ± 7.0	17 (94.4%)	NS	NS	16	18.1 ± 2.4	16 (100%)	NS	NS
Yoo et al., 2019 [43]	Korea	40	52.8 ± 4.3	25 (62.5%)	NS	40 (100%)	40	50.5 ± 7.0	27 (67.5%)	NS	40 (100%)
Zhang et al., 2020 [44]	China	28	62.3 ± 8.2	20 (71.4%)	NS	NS	27	61.6 ± 8.1	19 (70.4%)	NS	NS
Zhong et al., 2009 [45]	China	30	64 ± 8	17 (56.7%)	NS	NS	90	61.7 ± 8.3	56 (62.2%)	NS	NS

Legend: ASA: American Society of Anesthesiologists scale; BMI: body mass index; NS: not specified.

**Table 2:** Pooled analysis of adverse events between double-lumen tube and bronchial blocker groups.

Adverse Event	No. of Studies	Event/Participants		Events		Heterogeneity between Trials		*p*-Value for Differences across Groups
		DLT	BB	OR	95%CI	***p*** **-Value**	**I^2^ Statistics**	
Hypoxemia	6	30/223 (13.5%)	14/233 (6.0%)	2.27	1.14 to 4.49	0.74	0%	0.02
Hoarseness	9	76/301 (25.2%)	39/300 (13.0%)	2.30	1.39 to 3.82	0.32	14%	0.001
Sore throat	12	160/397 (40.3%)	106/455 (23.3%)	2.30	1.68 to 3.14	0.001	65%	<0.001
Lung infection	3	8/131 (6.1%)	7/129 (5.4%)	1.65	0.09 to 30.92	0.07	71%	0.74
Bronchus/carina injuries	3	22/95 (23.2%)	8/95 (8.4%)	3.45	1.43 to 8.31	0.26	25%	0.006

Legend: BB: bronchial blocker; CI: confidence interval; DLT: double-lumen tube; OR: odds ratio.

## Data Availability

The data supporting this study’s findings are available on request from the corresponding author (L.S.).

## Supplementary Materials

The following supporting information can be downloaded at: https://www.mdpi.com/article/10.3390/jcm12051877/s1, Table S1: A pooled analysis of baseline patients characteristics; Figure S1: A summary table of review authors’ judgements for each risk of bias item for each randomized study. Figure S2: A plot of the distribution of review authors’ judgements across randomized studies for each risk of bias item.
