# Supplementary material for: Systematic Review and Meta-Analysis of Efficiency and Safety of Double-Lumen Tube and Bronchial Blocker for One-Lung Ventilation
Source: J Clin Med. 2023 Feb 27;12(5):1877. doi: 10.3390/jcm12051877 (PMC10003923; doi:10.3390/jcm12051877)
Supplement: Supplementary file 1 [file jcm-12-01877-s001.zip › jcm-2192380-supplementary.pdf]

Systematic Review and Meta-Analysis of Efficiency and Safety of Double-Lumen Tube and Bronchial Blocker for One-Lung Ventilation

<u>Supplementary Digital File</u>

CONTENT:

Table S1: Pooled analysis of baseline patients characteristics 2

Figure S1: A summary table of re-view authors' judgements for each risk of bias item for each randomized study 3

Figure S2: A plot of the distribution of review authors' judgements across randomized studies for each risk of bias item. 4

Table S1: Pooled analysis of baseline patients characteristics

Adverse event	No. of studies	Event / Participants or MD (SD)		Events		Heterogeneity between Trials		p-Value for Differences across Groups
		DLT	BB	OR or MD	95%CI	p-Value	I^2 statistics	
Age	19	54.8 (12.9)	56.7 (12.8)	-0.23	-1.72 to 1.26	<0.001	71%	0.76
Sex, male	17	358/582 (61.5%)	392/648 (60.5%)	1.05	0.83 to 1.33	0.96	0%	0.69
Body mass index	10	26.2 (5.9)	25.8 (6.3)	0.30	-0.68 to 1.29	<0.001	71%	0.55
ASA								
1 class	8	81/323 (25.1%)	77/316 (24.4%)	1.09	0.71 to 1.65	0.60	0%	0.70
2 class	8	122/323 (37.8%)	121/316 (38.3%)	0.97	0.67 to 1.42	0.63	0%	0.89
3 class	8	108/323 (33.4%)	105/316 (33.2%)	0.97	0.57 to 1.66	0.86	0%	0.92
4 class	8	12/323 (3.7%)	12/316 (4.1%)	0.96	0.42 to 2.19	0.70	0%	0.92

Legend: ASA: American Society of Anesthesiologists classification; BB: bronchial blocker; CI: confidence interval; DLT: double lumen tube; MD: mean difference; OR: odds ratio

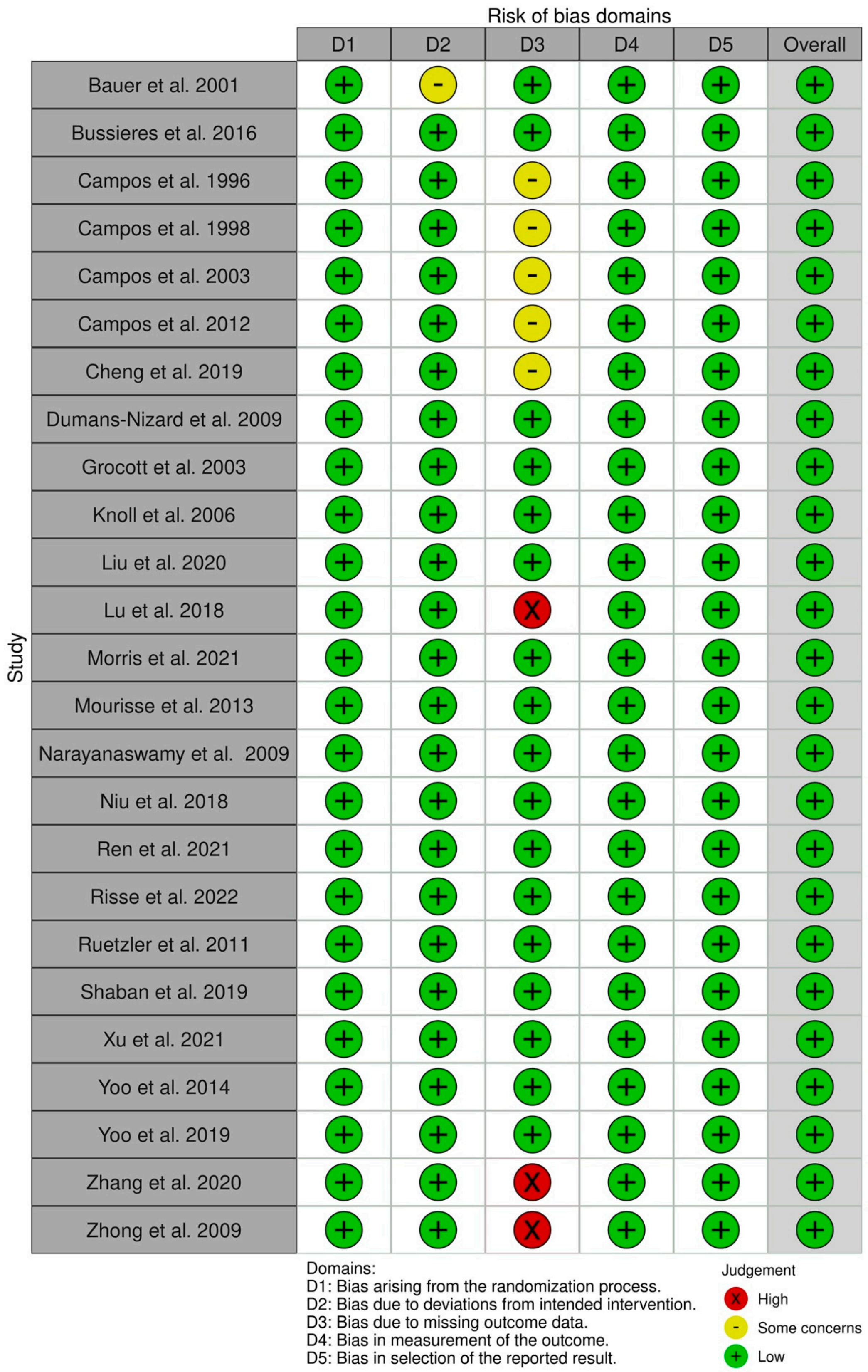

Figure S1: A summary table of re-view authors' judgements for each risk of bias item for each randomized study

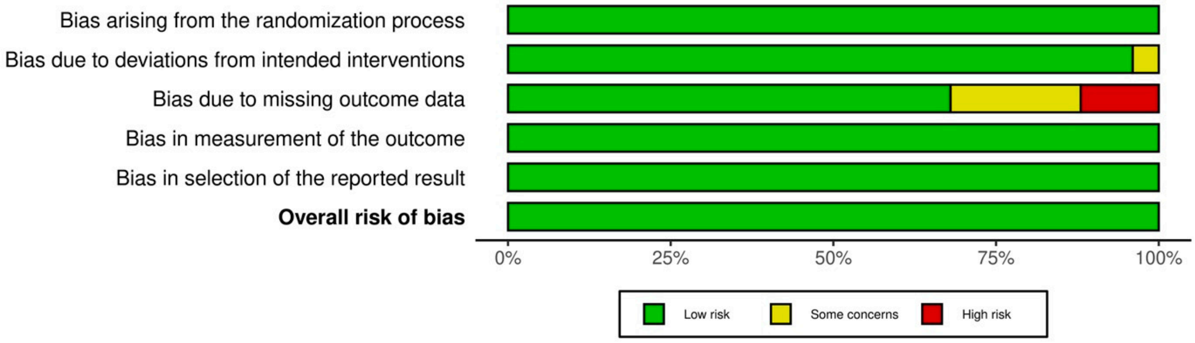

Figure S2: A plot of the distribution of review authors' judgements across randomized studies for each risk of bias item.
